# Machine Learning in Psychometrics and Psychological Research

**DOI:** 10.3389/fpsyg.2019.02970

**Published:** 2020-01-10

**Authors:** Graziella Orrù, Merylin Monaro, Ciro Conversano, Angelo Gemignani, Giuseppe Sartori

**Affiliations:** ^1^Department of Surgical, Medical, Molecular and Critical Area Pathology, University of Pisa, Pisa, Italy; ^2^Department of General Psychology, University of Padua, Padua, Italy

**Keywords:** machine learning, cross-validation, replicability, machine learning in psychological experiments, machine learning in psychometrics

## Abstract

Recent controversies about the level of replicability of behavioral research analyzed using statistical inference have cast interest in developing more efficient techniques for analyzing the results of psychological experiments. Here we claim that complementing the analytical workflow of psychological experiments with Machine Learning-based analysis will both maximize accuracy and minimize replicability issues. As compared to statistical inference, ML analysis of experimental data is model agnostic and primarily focused on prediction rather than inference. We also highlight some potential pitfalls resulting from adoption of Machine Learning based experiment analysis. If not properly used it can lead to over-optimistic accuracy estimates similarly observed using statistical inference. Remedies to such pitfalls are also presented such and building model based on cross validation and the use of ensemble models. ML models are typically regarded as black boxes and we will discuss strategies aimed at rendering more transparent the predictions.

## Introduction

The use of Machine Learning (ML) in psychometrics has attracted media attention after the Cambridge Analytica affair which dominated headlines around the world after the election of President Trump. Originally, academics from the Psychometric Centre from the University of Cambridge United Kingdom, collected a huge number of social media data (on over 50.000 participants) in order to predict personality of Facebook (FB) profile owners on the basis of their FB behavior. This research yielded a highly influential publication (Kosinski et al., 2013) were the authors showed how FB-based behaviors (i.e., likes) could be used to identify private traits with high accuracy (Christianity vs. Islam AUC = 0.82; Democrats vs. Republican, AUC = 0.88). Widespread attention arose because these data were opaquely leaked from the academic researchers to Cambridge Analytica, the now-infamous firm that scraped Facebook psychometric test data to construct millions of psychographic profiles, which it then used to hyper-target voters with custom-made campaign ads in favor of the Candidate Donald Trump during the presidential race of 2016. In short, Cambridge Analytica targeted “persuadable,” voters whose psychographic profiles (mostly a Big Five profiling) suggested they were open to suggestion.

A less media-attracting example of the use of ML in psychological science is the field of Psychometric Credit Score. A Psychometric Credit Score is a predictive model based on a microcredit applicant psychological and behavioral profile which is a substitute of the FICO score used for banked applicants, which, in turn is mainly based on bureau data and credit cards historical records (e.g., Meier and Sprenger, 2012). Fintech mobile apps powered by machine learning psychometric evaluations are testing microcredit applicants (e.g., for estimating the personal risk of the applicant) and are granted access to the data of the applicant’s smartphone which are fed into a machine learning model that extracts data relevant to the default prediction (e.g., number of phone calls during working hours is an indirect estimator of income, etc.). The psychological and behavioral data are used to estimate, using ML models, the default risk of the applicant and, for low risk applicants only, grant the loan asked for.

The above reported examples refer to the recent applications of ML and Deep Learning methods in psychological science that are emerging mainly outside the academic arena. However, the number of experiments reported in academic journals that use ML as analytical tools to complement statistical analysis is also increasing (Kosinski et al., 2013; Monaro et al., 2018; Pace et al., 2019). Machine learning has been successfully applied, for example, in the analysis of imaging data in order to classify psychiatric disorders (Orrù et al., 2012; Vieira et al., 2017), in genetics (Libbrecht and Noble, 2015; Navarin and Costa, 2017), in clinical medicine (Obermeyer and Emanuel, 2016), in forensic sciences (Pace et al., 2019) etc.

However, ML is not extensively used in the analysis of psychological experiments as compared to other fields (e.g., genetics). This seems particularly strange if we consider that mathematical modeling of cognitive/brain functioning had great advancements from psychology and neural network based cognitive modeling emerged as one of the main advancements in cognitive psychology (e.g., Seidenberg, 2005).

Experiments in psychological science has been traditionally analyzed with statistical inferential tools. However, recent controversies about the level of replicability in behavioral research of such analytical tools have cast interest in developing more efficient techniques for analyzing the results of psychological experiments (Pashler and Wagenmakers, 2012). ML has developed techniques that may control at least some forms of replicability, the replication of results with similar accuracy to unseen fresh new data.

### The Theoretical Role of Psychological Science in the Emergence of Machine Learning and Deep Learning

Hebb (1949) pioneered the mathematical modeling of a neural network that is still at the base of model based on reinforcement learning. He proposed what has come to be known as Hebb’s rule. He states, “*When an axon of cell A is near enough to excite a cell B and repeatedly or persistently takes part in firing it, some growth process or metabolic change takes place in one or both cells such that A’s efficiency, as one of the cells firing B, is increased.*”

Later, in 1958, Frank Rosenblatt, a Cornell psychologist (see Rosenblatt, 1962) in charge of The Perceptron Project designed what has been described as “*the first precisely specified, computationally oriented neural network*” (Anderson and Rosenfeld, 1988, p. 89).

Neural network modeling rebirth dated 1986 with the publication of David Rumelhart and Jay McClelland’s influential two−volume textbook, *Parallel distributed processing: Explorations in the microstructure of cognition, Volume 1: Foundations, Volume 2: Psychological and biological models, commonly referred to as the PDP Volumes*. In 1987, Walter Schneider noted that the Parallel Distributed Processing (PDP) volumes were already the basis for many courses in connectionism and observed that they were likely to become classics (Schneider, 1987, p. 77). His prediction was borne out. A leading figure in the group was Geoffrey Hinton, a Canadian psychologist turned-data-scientist who contributed to the first papers of the PDP group (McClelland et al., 1987), Hinton is now regarded as a godfather of deep and is now chief scientist at Google.

### Machine Learning in Analyzing the Results of Psychological Experiments

While psychology was in the front-end in theory building, is late in adopting ML as a tool for analyzing experimental results. In fact, psychological experiment results are largely analyzed by orthodox p-based statistical inference and more recently by effect size measures.

Here, we will not systematically review the recent advancement in modeling cognitive processes using ML/Deep Learning models (e.g., reinforcement learning) but rather focus on the benefits deriving from the more extensive use of ML methods in the analysis of results collected from psychological experiments as a complement to more traditional statistical inference techniques.

Here we claim that the use of ML could be a useful complement to inferential statistics and will help in achieving at least the following objectives:

– developing models which can generalize/replicate to fresh new data;
– developing models focused on prediction also at single subject level.

### The Difference Between Statistics and Machine Learning

In the now classic paper, Breiman (2001) highlighted the difference between statistical modeling and ML. He stated that the classical orthodox statistical approach assumes that data are generated by a given stochastic data mode and the evaluation is more focused on the degree of fitness that the data have to the model. Statistical inference based on data modeling has been the standard *de facto* procedure in the analysis of scientific experiments since 1940.

Inference creates a mathematical model of the data-generation process to formalize understanding or test a hypothesis about how the system behaves. Statistical methods have a long-standing focus on inference, which is achieved through the creation and fitting of a project-specific probability model. The model allows us to compute a quantitative measure of confidence that a discovered relationship describes a ‘true’ effect that is unlikely to result from noise. Measures typically include *p*-values with a recent shift to effect size in order to contrast the improper use of *p*-based inferences that may lead to a lack of replicability (Ioannidis et al., 2011).

By contrast, ML approach treats the data as unknowns and is mainly focusing on predictive accuracy. Prediction aims at forecasting unobserved outcomes or future behavior. Prediction is also addressed in statistics but with models that are usually constrained by strong assumptions (e.g., linear regression and logistic regression). ML models are more focused on prediction and “model agnostic.” It is a frequent observation that in most dataset analyzed with ML models similar predictions accuracies may be achieved using models that rely on very different assumptions (e.g., Support Vector Machine, Naive Bayes, Knn, Random Forest).

In ML models, prediction is achieved by using general-purpose learning algorithms to find patterns in often numerous and highly complex datasets.

ML methods are particularly helpful when one is dealing with datasets in which the number of input variables exceeds the number of subjects, as opposed to datasets where the number of subjects is greater than that of input variables.

ML makes minimal assumptions about the data-generating systems; they can be effective even when the data are gathered without a carefully controlled experimental design and in the presence of complicated non-linear interactions. However, despite convincing prediction results, the lack of an explicit model can make ML solutions difficult to directly relate to existing biological knowledge.

The boundary between statistical inference and ML is fuzzy and methods originally developed in statistics are included in the ML toolbox. For example, logistics among classifiers, linear regression among regression techniques, hierarchical clustering among clustering techniques and Principal components analysis among dimensionality reduction techniques are routinely included in all ML packages. Some of these models (e.g., logistics) usually compares favorably with more complex models (Zhang et al., 2019) with respect to accuracy.

Statistics requires us to choose a model that incorporates our knowledge of the system, and ML requires us to choose a predictive algorithm by relying on its empirical capabilities. Justification for an inference model typically rests on whether we feel it adequately captures the essence of the system. The choice of pattern-learning algorithms often depends on measures of past performance in similar scenarios. Inference and ML are complementary in pointing us to biologically meaningful conclusions.

The agnostic empirical approach of ML is best understood considering the Naive Bayes classifier. The Naive Bayes algorithm is an intuitive method that uses the probabilities of each feature (independent variable) predicts the class the individual case belongs to. It is referred to as “naive” because all features are regarded as independent, which is rarely the case in real life. Naive Bayes simplifies the calculation of probabilities by assuming that the probability of each attribute belonging to a given class is independent of all other attributes. This is a strong and frequently false assumption but results in a fast and effective classification method. Despite the apparently unrealistic assumptions it has been shown the mathematical properties of the good performance of the classifier (Ng and Jordan, 2001). It has been shown that no matter how strong the dependencies among attributes are, Naive Bayes can still be optimal if the dependencies distribute evenly in classes, or if the dependencies cancel each other out (Zhang, 2004). Basically, Naive Bayes is finding the probability of given feature being associated with a label and assigning the label with the highest probability. Despite the assumption of independence the Naive Bayes classifier is usually performing well and is used in practice for a number of practical reasons (e.g., no need to handle inter-correlations, small computational time, performs well for categorical input data, needs less data with respect to other classifiers, e.g., logistics). The success of Naive Bayes classifier is an example of the empirical approach that is characterizing ML modeling. What counts is predictive efficiency rather than how well-prediction based on correct assumptions reliably approximate the data. We will see, in the simulation reported below, that Naive Bayes results among the best classifiers and among those that consistently generalizes across different datasets.

### Machine Learning Models

ML models are typically distinguished in supervised models and unsupervised models. Supervised models are built from examples which are labeled. By contrast unsupervised models are developed using unlabeled examples and consists in grouping examples on the basis of their similarities (e.g., clustering, anomaly detectors, etc.) (Mohri et al., 2012).

Supervised models may be further distinguished in classifiers and regressors. Classifiers deal with classification problems when the output variable is a category (e.g., “disease” vs. “no disease”). Regressors address regression problems when the output variable is a real value (e.g., Reaction Time).

Some ML learning models deal only with classification problems (e.g., Naive Bayes) while others may be used both for classification and regression (e.g., Decision trees, Artificial neural Networks, Random Forest) and their use depends on the problem that is addressed.

Here, we will focus on supervised models used for classification among which we could list:

(1) **Decision Trees:** decision tree builds classification or regression models in the form of a tree structure. It utilizes an if-then rule set which is mutually exclusive and exhaustive for classification. The rules are learned sequentially using the training data. Each time a rule is learned, the tuples covered by the rules are removed. This process is continued on the training set until meeting a termination condition. The tree is constructed in a top-down recursive divide-and-conquer manner. Simple decision trees have the advantage of transparency as the final user understands the prediction rules. However, complex decision tree models such as Random forest (e.g., Breiman, 2001) and Xgboost usually outperform the most simple decision trees.
(2) **Naive Bayes:** Naive Bayes (John and Langley, 1995) is a probabilistic classifier inspired by the Bayes theorem under a simple assumption which the attributes are conditionally independent. Even though the assumption is not valid in most cases since the attributes are dependent, surprisingly Naive Bayes performs impressively in a variety of datasets.
(3) **Artificial Neural Network (ANN)**: is a brain-inspired model with a set input/output units where each connection has a weight associated. ANNs were originally developed by psychologists and neurobiologists to develop and test computational analog of neurons. During the learning phase, the network learns by adjusting the weights (strength of the synapses of the virtual neuron) so as to be able to predict the correct class label of the input stimulus. ANN could be used both for classification and regression.
(4) **k-Nearest Neighbor**: is a lazy learning algorithm which stores all instances in a n-dimensional space. When an unknown new data must be classified, it analyses the closest k number of instances saved (nearest neighbors) and returns the most common class as the prediction. In the distance-weighted nearest neighbor algorithm, it weighs the contribution of each of the k neighbor’s according to their distance using the giving greater weight to the closest neighbors (Aha et al., 1991). KNN could be used both for classification and regression.
(5) **Logistic Regression:** (Le Cessie and van Houwelingen, 1992) is a powerful statistical way of modeling a categorical outcome with one or more explanatory variables. It measures the relationship between the categorical dependent variable and one or more independent variables by estimating probabilities using a logistic function, which is the cumulative logistic distribution.
(6) **Ensemble Methods:** are learning algorithms that construct a set of classifiers and then classify new data points by taking a weighted vote of their individual predictions. The original ensemble method is Bayesian averaging, but more recent algorithms include error-correcting output coding, bagging, and boosting. Ensemble models, by combining different classifiers, usually perform better with a reduction of prediction variability when compared with their constituent classifiers. Ensemble methods usually outperform single classifiers as can be seen in Kaggle competition winners solutions. Ensemble methods usually are optimal solutions of the so called bias/variance trade-off. Usually Bias, the amount of systematic error in prediction, is related to the complexity of the model and highly complex models tend to have low bias but also overfit (e.g., Random Forest). By contrast, simple models, which make few assumptions, tend to underfit. Variance refers to the variability in the predictions, which is usually high in complex models and low in simple models.

There are two procedures that in some cases may enhance a classifier performance apart of ensembles models: feature selection and feature engineering and parameter tuning. Feature selection consists in selecting among the all features (independent variables) the most informative ones while feature engineering consists in deriving new features usually basing on domain knowledge and preliminary data analysis. In other words, feature engineering is about creating new input features from existing ones with the intention to boost the performance of ML models. In psychological test development, feature selection and engineering may be used to derive a subset of items (e.g., the original tests) that performs similarly to the full test and eventually enhance efficiency via developing combination of features.

Parameter tuning consists in selecting the optimal value for parameters of the model that are intended to be used. For example Knn, is a classification model with a single parameter which is the number of neighbors that are used to decide the category of which the new example belongs to. The winning class that is assigned to the new unlabeled case will result from computing the majority of neighbors. The dimension of the neighborhood (2, 3…10, 11) is a parameter that may be optimized and identified as the one that gives maximum performance. In some cases, such as in deep learning models of object detection, the number of parameters to be estimated is in the order of 100.000.

### The Interpretability/Accuracy Trade-Off

Best performing models are usually hard to interpret giving rise to a clear interpretability/accuracy trade-off (Johansson et al., 2011). For example, Fernández-Delgado et al. (2014) evaluated the performances of 179 ML classifiers on 121 different datasets arriving to the conclusion that the best performer is Random Forest with support vector machine (SVM) notably second (no significant difference between the two). Additional investigations (Wainberg et al., 2016) re-analyzing the data claimed the Random Forest superiority was not significantly better than SVM and Neural Networks. However, for what counts here, Random Forest, as well as Neural Network and SVM are all hard to interpret. Simpler models, such as pruned decision rules (J48), Naive Bayes, Knn are easier to interpret but rarely result in having the best performance.

Some insight on the interpretability/accuracy trade-off may also come from inspecting the strategies used by Kaggle masters. Kaggle is a site where ML experts can compete in finding the best predictive model on a public dataset. The Netflix Prize was one of these competitions (prize $100.000). Best practices collected from such ML competitions indicate that winners systematically rely on the following strategies in deploying winning models: (i) feature engineering (finding new features usually combinations of the available ones), (ii) parameters tuning (finding the optimal parameters of the model that maximize performance), and (iii) ensemble learning (build a complex model which is a combination of more simple models). Ensemble learning performs better than the constituent classifiers but this reduces interpretability. An example is the difference in the interpretability of a single decision rule when contrasted with a random forest model on the same data. The single decision rule is transparent (e.g., *if X* > *3.5 than class A else B*) while Random Forest (of decision rules) results in an uninterpretable random mixture of a high number (e.g., 100) decision rules that render opaque any understanding on the exact mechanism at the base of prediction.

In short, interpretable models usually are not the best performers and the best performers classifiers are usually not interpretable. This means that using ML models for analysis results of psychological experiments one could use hard-to-interpret ensemble models to have an estimate of the maximum accuracy possible while using easy-to-interpret decision rules for more confidence based evaluations.

### Replicability of Results and Cross Validation

The recent focus on the lack of replicability in behavioral experiments is known with the term of replicability crisis. One source of potential problem leads back to the use of inferential statistics and its misunderstanding of *p*-values and underpowered experiments (Baker, 2016). Recent methodological discussions are related to procedures that guarantee replicable results (Browne, 2000). In summarizing their assessment of replicability Szucs and Ioannidis (2017) concluded that:

“*Assuming a realistic range of prior probabilities for null hypotheses, false report probability is likely to exceed 50% for the whole literature. In light of our findings, the recently reported low replication success in psychology is realistic, and worse performance may be expected for cognitive neuroscience.*”

Replication of experimental results may be distinguished in exact and broad replication (Cumming, 2008). Exact replication refers to a replication using exactly the same procedure of the original experiment and is targeted by cross validation. The author (Cumming, 2008) proved, in a simulation study of 25 repetitions, that a result in the first experiment significant at *p* < 0.05 in the replications may vary from *p* < 0.001 to *p* = 0.76 (with a 10% chance of *p* > 0.44) showing that p is a very unreliable measure. To complicate the landscape, some researchers have also highlighted how failed replication are not immune from the same type of error that may be detected in the original studies (Bressan, 2019) and false negatives in replication studies have recently attracted attention (Bryan et al., 2019).

Similarly to analysis conducted with inferential statistics, ML workflow encounters the problem replication (Gardner et al., 2018; Gundersen and Kjensmo, 2018). In fact, it is easy to develop complex ML models (e.g., Random Forest) that on small datasets reach near perfect classification accuracies (McDermott et al., 2019). However, this accuracy does not replicate to fresh data which are not used to develop the model (holdout data). For this reason a *de facto* standard for handling this overfitting problem, that plagues not only ML models but also statistical models (e.g., logistics, linear regression) is cross validation.

**FIGURE 1:**
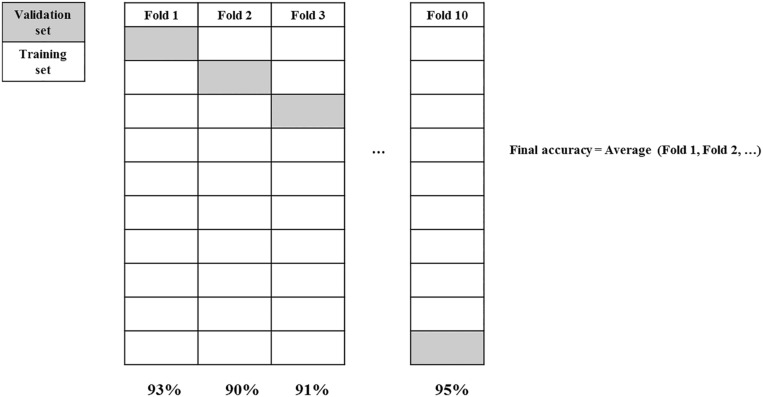
10-fold cross validation.

Cross Validation (see Figure 1) is usually a very good procedure to measure how well a result may be replicable at least for what has been called exact replication (Cumming, 2008). Even if ideally it does not address reproducibility of the main finding when minor variations are introduced, exact replication refers to replication where all the conditions of the original experiment are maintained. As cross validation consists in evaluating models on a hold-out set of experimental examples, this set do not differ from the examples used for model development. While cross validation does not prevent the model to overfit, it still estimates the true performance.

In order to avoid overfitting, cross validation regards a compulsory step in ML analysis but its use is very limited in the analysis of psychological experiments. There are a number of different cross validation procedure but one which guarantees good result is the so called stratified 10-fold cross validation. In order to develop models able to generalize new data (unseen data) a good procedure envisages to: (1) remove the 20% of the data for validation; (2) run 10-fold cross validation on the remaining 80% with the aim to select optimal parameters; (3) train model with all 80% of the data with optimal parameters; (4) test the model on the 20% validation set. The result of step 4 will be the best approximation to exact replication of the experiment.

A special case of n-fold cross validation is the LOOCV (Cawley and Talbot, 2010) a method of choice in imaging studies with clinical samples (Orrù et al., 2012). In LOOCV, the statistical model is developed using only n-1 examples and tested on the remaining one exemplar. The procedure is repeated rotating systematically the left out case and the final out-of-sample classification error estimate is derived from the average error of the n-1 models.

When running a cross validation, special care is needed to control information leakage which is one of the reasons why cross validation goes wrong. For example, selecting a subset of predictors before cross validation is a form of leaking knowledge that reduces generalization.

Most psychometric investigations do not address the problem of generalization outside the sample used to develop the model. Clearly, avoiding cross validation yields inflates results, which are over optimistic and may not replicate when the model is applied to out-of-sample data. Similar results have been recently reported by Bokhari and Hubert (2018). The authors reanalyzed the results of the MacArthur Violence Risk Assessment Study using ML tree models and cross validation. Also Pace et al. (2019), in discussing the results of the B test (a test for detecting malingered cognitive symptoms), similarly observed that a decision rule developed on the whole dataset yielded a classification accuracy of whole dataset 88% but using LOOCV the expected accuracy drops to 66%.

### Working Example: ML Analysis on Millon Clinical Multiaxial Inventory (MCMIIII)

The example below (Table 1) regards the psychometric identification of malingering (Sartori et al., 2016, 2017). The dataset analyzed here consists in the raw scores on the personality questionnaire MCMI-III that was used to predict whether the test was collected in one of two settings. Both groups are low credibility groups, the first are fake good suspects (they have advantages from denying psychopathology) while the second are fake bad suspects (they have advantages from doctoring a get-out-of-jail psychopathology). One group was administered the test for a psychological assessment for reinstatement of driving license and child custody court assessment (*n* = 93) while the fake bad group included cases involved in a criminal trial who underwent a mental insanity assessment (*n* = 93). Input were a total of 27 MCMI-III scores, which were used to predict whether the test results were drawn from a Fake good setting or Fake bad, setting. To check the level of replicability, models were tested on 62 new cases extracted, as a first step of the procedure, from the original sample of 186 + 62 cases^1^.

**TABLE 1:** ML analysis conducted on 186 participants tested with the MCM III.

			**Stratified**	**Model overfitting**
		**Cross**	**holdout**	**training *minus***
	**Training set**	**validation**	**test set**	**stratified holdout**
**Classifier**	**(n = 186)**	**(n = 186)**	**(n = 62)**	**test accuracy**
Naive Bayes	67%	65%	66%	1%
Logistic	75%	62%	58%	17%
SVM	74%	70%	67%	7%
Knn	79%	70%	64%	15%
OneR	70%	62%	67%	3%
CART	93%	62%	61%	32%
Random forest	100%	66%	64%	36%
Neural network	96%	66%	69%	27%
(Averaging)	81.6%	65.4%	65.3%	0.1%
	(12.7)	(3.33)	(3.37)	
Ensemble learner	80.6%	67.7%	69.4%	1.7%

*Half of the participants belonged to the Fake-Good group and Half to the Fake-Bad group. A stratified holdout test set (*n* = 62) was used to evaluate the generalization/replicability of the predictions. Note how the 10-fold cross validation conducted on the training data (*n* = 186) is a good approximation of the performance on the holdout test set (*n* = 62). The ensemble model performed slightly better than the performance of the constituents ML models.*

As seen above, if a model is developed on all the available data then the final accuracy will be an over optimistic estimate that is not confirmed when the model is tested on previously unseen data (out-of-sample dataset).

From the inspection of the above reported table it appears that:

– Developing the model on all the available training data leads to an accuracy which is not replicated on the test set (average; 81.6 vs. 65.3%).
– The 10-fold cross validation leads to accuracy estimates that correspond to that obtained on the test set. Exact replication on the test set show that the 10 fold cross validation does not lead to an overly optimistic estimate. In short, models developed with cross validation replicate well (see also Koul et al., 2018).
– There is no clear winner among the classifiers. Very simple classifiers (in terms of parameters that require estimation) give comparable results to more complex models (compare Naive Bayes and Knn versus Random Forest and Multilayer Neural Network).
– The ensemble of many classifiers performs well on new data and therefore replicates well on fresh new data.
– Some very complex models with many parameters to estimate show extreme overfitting (Random Forest and Multiplayer Neural Network). For example, a Random Forest model developed on the training set yielded a perfect classification (100% accurate) while after a 10-fold cross validation accuracy drops to 66 and 64% on the stratified holdout test set. On the same data the figures for a Multilayer Neural network were 96% on the total of the sample while the result drops to 66% after a 10-fold cross validation which approximates well the 69% measured on the holdout test set. Cross validation is therefore approximating results in exact replication with high accuracy.
– Some very simple models (Naive Bayes) do not suffer much from overfitting when trained without cross validation.
– Also decision rules (usually developed in psychological test building for identifying test cut-off) when fine-tuned without cross validation may heavily overfit. Note that decision rules (e.g., OneR) are the method of choice in most neuropsychological and personality tests; they are simple, readily interpretable but they also need cross validation because they also suffer from overfitting and low replicability.

As regards to exact replicability, it has been noted that results, analyzed with statistical inferences techniques, when replicated show a reduced effect size. In short, an original experiment with an effect size of *d* = 0.8 when replicated shows an effect size *d* = 0.4. Repeated K-fold cross validation may derive a distribution of measures generalization/replication.

### Characteristics of the Dataset

High performance neural networks are trained with extremely large dataset. For example a deep neural network with 152 layers and trained on a Imagenet dataset (*n* = 1.2 mn images) has reduced to 3% the error in classifying images (He et al., 2016). It has been well-established that for a given problem, with large enough data, very different algorithms perform virtually the same.

However, in the analysis of psychological experiments typical number of data points is in the 100 range. Do ML classifiers trained on such small dataset maintain their performance?

In order to evaluate the capacity of ML models to replicate classification accuracies on small datasets, we ran a simulation using the dataset used for the simulations reported in Table 1. A total of 298 participants assessed in a low credibility setting (124 in the fake good group and 124 in the fake bad group) were administered the MCMI-III as a part of a forensic assessment. The whole dataset was split into four stratified subsets (folds). Each ML model was trained on one of these folds (using 10-fold cross validation) and tested on the remaining three. The results are reported in Table 2.

**TABLE 2:** Different machine learning models trained using 10-fold cross validation.

				**Max% diff**
**CV on Fold**	**Tested on**	**Tested on**	**Tested on**	**(average = 8.3%)**
**(a) Naive Bayes**				
Fold 1 = 68%	Fold 2 = 73%	Fold 3 = 68%	Fold 4 = 66%	5%
Fold 2 = 69%	Fold 1 = 65%	Fold 3 = 66%	Fold 4 = 66%	4%
Fold 3 = 69%	Fold 1 = 63%	Fold 2 = 73%	Fold 4 = 66%	6%
Fold 4 = 65%	Fold 1 = 65%	Fold 2 = 66%	Fold 3 = 63%	2%
**(b) SVM**				
Fold 1 = 63%	Fold 2 = 70%	Fold 3 = 71%	Fold 4 = 69%	8%
Fold 2 = 69%	Fold 1 = 66%	Fold 3 = 66%	Fold 4 = 61%	8%
Fold 3 = 69%	Fold 1 = 70%	Fold 2 = 69%	Fold 4 = 61%	8%
Fold 4 = 65%	Fold 1 = 74%	Fold 2 = 67%	Fold 3 = 72%	9%

				**Max% diff**
**CV on fold**	**Tested on**	**Tested on**	**Tested on**	**(average = 9.5%)**

**(c) Random forest**				
Fold 1 = 62%	Fold 2 = 69%	Fold 3 = 67%	Fold 4 = 58%	7%
Fold 2 = 72%	Fold 1 = 66%	Fold 3 = 64%	Fold 4 = 67%	8%
Fold 3 = 71%	Fold 1 = 69%	Fold 2 = 67%	Fold 4 = 56%	15%
Fold 4 = 63%	Fold 1 = 66%	Fold 2 = 71%	Fold 3 = 64%	8%

				**Max% diff**
**CV on fold**	**Tested on**	**Tested on**	**Tested on**	**(average = 7%)**

**(d) Ensemble**				
Fold 1 = 65%	Fold 2 = 67%	Fold 3 = 69%	Fold 4 = 61%	5%
Fold 2 = 69%	Fold 1 = 64%	Fold 3 = 65%	Fold 4 = 63%	6%
Fold 3 = 68%	Fold 1 = 65%	Fold 2 = 74%	Fold 4 = 60%	8%
Fold 4 = 63%	Fold 1 = 71%	Fold 2 = 69%	Fold 3 = 72%	9%

*Results are reported on testing a ML model on each of the four stratified folds (using 10-fold cross validation) and tested on each of the remaining 3. Results of three classifiers are reported as well as the results of an ensemble model built using all the classifiers included in Table 1. The maximum error is reported as well as the average error%. *CV*, cross validation.*

As shown in Table 2 all the classifiers trained on a small dataset of 62 cases (32 per each of the two categories) perform well on each of the other test folds. Simple classifiers (e.g., Naive Bayes) perform slightly less erratically across holdout folds than more complex one (e.g., Random Forest). A good strategy in developing ML models that replicates well is to train simple classifiers or ensemble of classifiers rather than models with many parameters.

### Balanced Versus Unbalanced Datasets and Priors

In all the examples reported above the number of cases for each class was equal. Unbalanced datasets are usually a problem for classifiers and usually performance of classifiers is generally poor on the minority class. For this reason a number of techniques have been developed in order to deal with unbalanced datasets.

Another problem often neglected is that the final accuracy is the result not only of the accuracy of the model but also depends on the prior probability of the class under investigation. For example, if the prior probability of the class is 10% and the accuracy of classifiers trained on a balanced dataset is 90% the actual probability that a case is correctly classified in the minority class is 50% (of the 18 classified 9/18 will be correct).

### Comparing Statistical Inferences With Machine Learning Results

ML uses evaluation metrics mainly addressing accuracy in classification such as Accuracy, area under the curve (AUC), etc. By contrast, statistical metrics are different and more linked to inference (*p*-values) and more recently focusing on reporting effect sizes (e.g., Cohen’s d etc.).

One problem that requires to be addressed when complementing statistical analysis with ML results is in the comparison between the metrics used in statistics (e.g., r, d, etc.) and the typical metrics used in ML (classification accuracy, F1, AUC).

Salgado (2018) addressed the problem of translating performance indicators from ML metrics and statistical metrics. It has been shown that the most used ML evaluation metrics can be mapped into effect size; for example, it has been shown that an AUC = 0.8 corresponds to a Cohen’s *d* = 1.19. It is possible to transform the accuracy results obtained from ML models to more psychologically oriented effect size measures (Salgado, 2018). It is worth noting, that a Cohen’s *d* of 0.8 is usually regarded as large but, when translated to classification accuracy among two categories, corresponds to an accuracy in classification of 71% due to an overlap between the two distributions of 69%. Using results from Table 1 an out-of-sample accuracy of 65.3% resulting from the averaging of various classifiers corresponds to a Cohen’s *d* = 0.556, usually regarded as a medium effect (Cohen, 1977). However, an accuracy of 65.3% in distinguishing fake good versus faked bad responders of MCM III is far from being of any practical utility when applying the test at single subject level (as in clinical usage of the test).

### Model-Hacking in Machine Learning

One procedure which is believed to be at the origin of lack of replicability in reporting experimental results, analyzed with statistical inference, is the so called p-hacking (Nuzzo, 2014).

In ML analyses, there is a similar source of lack of replicability, which could be called model hacking. If many models are tested in order to report only the best model, we are in a condition similar to p- hacking. In the example reported in Table 1, using cross validation and reporting only the best performer among the classifiers, in this case SVM, would have produced an accuracy estimation in excess of 4.5% (SVM cross validation results = 70%; average of all cross validation results = 65.5%).

In order to avoid model hacking, one strategy is to verify that classification accuracy is not changing much among different classes of classifiers (see Monaro et al., 2018) as follows: if similar results are obtained by ML models relying on radically different assumptions, we may be relatively confident that the results are not dependent on such assumptions. Additionally, model stability may be addressed by combining different classifiers into an ensemble classifier that indeed reduces the variance in out-of-sample predictions and therefore gives more reliable predictions. Using ensembles instead of specific classifiers is a procedure that avoids model-hacking.

## Conclusion

Academic psychologists have pioneered the contemporary ML/deep learning development (Hebb, 1949; Rumelhart et al., 1986) and cognitive theorists used connectionist modeling in the field of reading, semantics, attention (Seidenberg, 2005) and frequently anticipated the now much spoken about technology advancements in such fields such as Natural Language Processing (e.g., Word2vec and Lund and Burgess, 1996) and object recognition.

By contrast, ML/deep learning models used for cognitive theorizing have been rarely used in the analysis of psychological experiments and in psychometric test development (Mazza et al., 2019). Classification of brain images (both functional and structural) is a notable exception (Orrù et al., 2012; Vieira et al., 2017).

We have highlighted, in this paper, the reasons why ML should systematically complement statistical inferential analysis when reporting behavioral experiments. Advantages derived from using ML modeling in an analysis experimental results include the following:

– generalization/replication of results to unseen data is realistically estimated rather than optimistically inflated;
– n-fold cross validation guarantees replicable results also for small datasets (e.g., *n* = 40) which are typical in psychological experiments;
– practical and clearly understandable metrics (e.g., out-of-sample accuracy) are reported, rather than indirect inferential measures;
– personalized predictions at single subject level (specific single subjects estimations may be derived also when there are numerous predictors) and subjects which are classified erroneously may be individually analyzed;
– more realistic estimate about the utility of a diagnostic procedure.

Known potential pitfalls of ML data analysis that may obstacle a more extensive use of the ML methods are:

– model hacking. When only the single best performer model is reported rather than a variety of models with differing theoretical assumptions. Model hacking may lead to an overestimation of replicable results. A remedy against model hacking consists in reporting many ML models or ensemble models;
– lack of interpretability. Usually maximum accuracy in prediction is achieved with highly complex non-interpretable models such as XGboost, Random Forest and Neural Networks. This is probably the single most important problem in clinical applications where the clinician needs a set of workable rules to drive the diagnosis. To tamper the problem it may be useful to report simple decision rules that may help in evaluating the cost of non-interpretability (accuracy achieved with simple interpretable models as compared to maximum accuracy achieved by complex less interpretable models). Interpretability is important in clinical setting where clinicians need simple and reliable decision rules (see Figure 3 in Mazza et al., 2019).

## Data Availability Statement

The datasets analyzed in this article are not publicly available. Requests to access the datasets should be directed to giuseppe.sartori@unipd.it.

## Author Contributions

GO devised the main research topic, and planned and carried out the ML analysis. GO, MM, and GS conceived the conceptual ideas and proof outline. GO, MM, CC, AG, and GS drafted the manuscript, revised the manuscript critically, and gave the final approval for the version to be published.

## Conflict of Interest

The authors declare that the research was conducted in the absence of any commercial or financial relationships that could be construed as a potential conflict of interest.

## Footnotes

1 All the analysis reported here were done in WEKA (Hall et al., 2009), one of the open sourced code-free ML tools available. Other no-code GUI-based software packages are: ORANGE, KNIME and for deep learning Uber’s LUDWIG.
